# Early genetic events in the colorectal carcinogenic pathway of familial adenomatous polyposis and sporadic polyp: germline and somatic alterations in carcinogenesis

**DOI:** 10.3389/fgene.2025.1668133

**Published:** 2025-12-19

**Authors:** Hiroki Tanabe, Yusuke Mizukami, Yusuke Ono, Hidehiro Takei, Nobue Tamamura, Yu Kobayashi, Keitaro Takahashi, Katsuyoshi Ando, Nobuhiro Ueno, Shin Kashima, Mishie Tanino, Kentaro Moriichi, Mikihiro Fujiya, Toshikatsu Okumura

**Affiliations:** 1 Division of Gastroenterology, Department of Internal Medicine, Asahikawa Medical University, Asahikawa, Hokkaido, Japan; 2 Institute of Biomedical Research, Sapporo-Higashi Tokushukai Hospital, Sapporo, Hokkaido, Japan; 3 Department of Diagnostic Pathology, Asahikawa Medical University Hospital, Asahikawa Medical University, Asahikawa, Hokkaido, Japan

**Keywords:** FAP (familial adenomatous polyposis), NGS-next-generation sequencing, cancer, adenoma, APC

## Abstract

**Purpose:**

Genetic mutations in the tumor suppressor gene *APC* and the oncogene *KRAS* are an initial event in the colorectal adenoma-carcinoma sequence. Multistep carcinogenesis has been discovered through the study of familial adenomatous polyposis (FAP), an inherited disease with a germline *APC* variant. We aimed to determine the premalignant mutational genotypes that progress to colorectal neoplasia using target sequencing to compare the characteristics of FAP patients with sporadic cases.

**Experimental design:**

A total of 197 samples from 20 FAP and 13 sporadic cases were analyzed using next-generation sequencing (NGS) with a cancer panel. The analysis of *APC* germline variants identified FAP patients with a germline variant, those with whole APC deletion, and those with no alterations. The association between pathogenic germline variants and somatic mutations was assessed.

**Results:**

Colorectal tumors of FAP and non-polyposis patients showed a similar frequency of mutations (*APC,* 76% and 75%; *KRAS*, 32% and 25%). Somatic *APC* mutations in FAP patients was observed in the mutation cluster region (63.3%). In FAP, many colorectal tumors (57.5%) harbored two *APC* hits, whereas in sporadic cases, one or two hits were more common (44.4% and 22.2%, respectively). Of the 99 tumors in FAP patients with *APC* germline variants as the first hit, 74 tumors (74.7%) acquired somatic mutations as the second hit, and 9 tumors (9.9%) further gained a third hit, indicating a ‘three-hit’ alteration.

**Conclusion:**

An identical cancer pathway may be associated with multistep carcinogenesis, accompanied by *APC* mutations in mutation hotspots. A combined analysis of germline and somatic alterations revealed ‘three-hit’ alterations in the *APC* gene among the FAP patients, suggesting that the heterogeneity of colorectal carcinogenesis contribute to these genetic changes.

## Introduction

Colorectal carcinoma (CRC) develops through a process termed the adenoma-carcinoma sequence, which refers to a stepwise process of mutational activation of oncogenes (Vogelstein et al., 1998; Kinzler and Vogelstein, 1996). Adenomatous polyposis coli (*APC*) mutations are most frequently observed in the early stages of colorectal carcinogenesis. Additional genetic mutations, including *CTNNB1, FBXW7, KRAS*, *BRAF, PIC3CA*, *p53,* and *SMAD4,* accumulate during CRC development (Jones et al., 2008). The Cancer Genome Atlas (TCGA, 2012) has increased our understanding of the pathogenesis of CRC, in which certain driver mutations are identified under malignant conditions.

Familial adenomatous polyposis (FAP) is an inherited disease characterized by the development of more than 100 adenomatous polyps in the large intestine. Germline variants in *APC* are inherited from parents or sometimes occur spontaneously. *APC*, which is located on chromosome 5q, encodes 2843 amino acids. APC is a component of the Wnt signaling pathway and plays a role in intercellular adhesion (Miyaki et al., 2008). Germline pathogenic variants are distributed widely from exons 1 to 15, and genotype-phenotype correlations are observed depending on the location of the variants (Miyoshi et al., 1992). The typical form of FAP with profuse polyposis is associated with variations in the mutation cluster region (MCR) between codons 1,286 and 1,513 (Kohler et al., 2008). Somatic *APC* mutations in colorectal cancers are typically nonsense variants and insertion and/or deletion mutations that result in amino acid truncation. *APC* mutations within the MCR produce truncated APC proteins that lack all axin-binding sites and all but 1 or 2 of the 20 amino acid repeats (20AARs) (Kohler et al., 2008). Patients with germline mutations after codon 1,399, which retain 2 to three intact 20AARs, acquire truncating mutations in the MCR. Conversely, patients with germline mutations to the MCR tend to have somatic mutations in the MCR and retain 1 to 2 20AARs (Albuquerque et al., 2002). This suggests that second hits in *APC* are selected to produce a ‘just-right’ level of β-catenin signaling optimal for colorectal tumor development. The combined hits resulting in partial loss of β-catenin regulation are suggested as a loose fit model (Crabtree et al., 2003). Studies of adenomas from patients with attenuated FAP have further revealed third hits in *APC* targeting the germline mutant allele to achieve an optimal genotype. Since these extensive studies concerning the two-hit hypothesis for colorectal tumorigenesis were conducted in the late 20th century, Sanger sequencing was used to identify germline variants in patients with polyposis. Next-generation sequencing (NGS) has been conducted for large scale regional analyses (Takao et al., 2021; Torrezan et al., 2013). However, few studies have investigated the molecular alterations in early precancerous colorectal tumors of FAP patients, compared with sporadic tumors using NGS.

We explored molecular alterations using target sequencing in the early phase of adenoma-carcinoma sequences and analyzed the *APC* gene alterations in these early lesions by comparing colorectal neoplasms in FAP patients with sporadic colonic neoplasms.

## Materials and methods

### Study population

The study participants were recruited from Asahikawa Medical University between 2018 and 2024. Potentially eligible FAP patients met one of the following criteria: (1) molecular diagnosis, (2) clinical diagnosis. The patient with <100 adenomas was defined as attenuated FAP (AFAP), and the presence of numerous adenomas rendering the normal mucosa in the entire colon invisible was defined as the profuse type. The remaining cases were defined as the sparse type. Twenty FAP patients underwent surgical resection or endoscopic resection. The tissues were fixed in formalin and paraffin embedded for histological diagnosis. To confirm germline variants in some cases, blood was used for Sanger sequencing or array comparative genomic hybridization (aCGH). For analysis of the conventional colorectal adenoma-carcinoma sequence, non-polyposis patients with sporadic colorectal polyps that were endoscopically resected were randomly selected from the medical records in the endoscopy division. Thirteen patients with colorectal polyps were included in this study.

**FIGURE 1:**
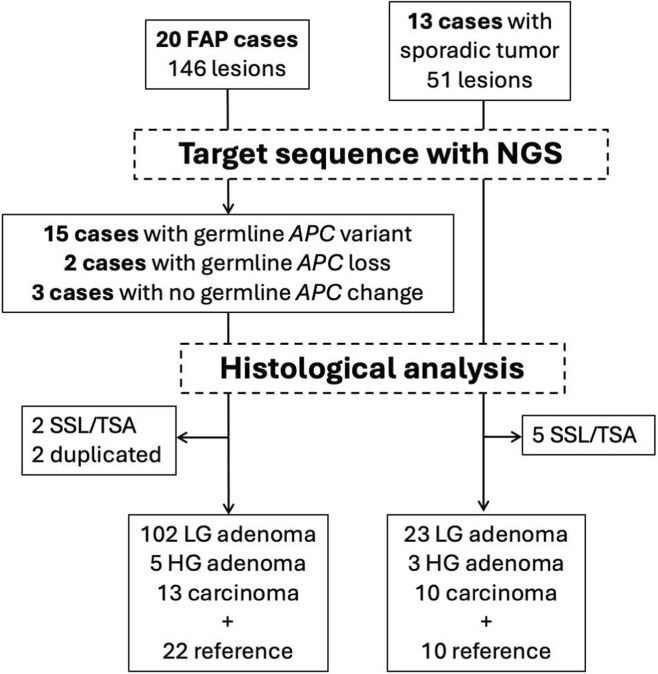
The genetic analysis of colorectal tumors. Flow charts of sample enrollment in the study. Familial adenomatous polyposis (FAP) patients and non-polyposis patients with sporadic tumors are enrolled in this analysis. FAP patients are classified due to germline variant into three groups: *APC* variant, *APC* loss, no *APC* change.

Samples from 197 lesions were subjected to NGS (Figure 1). Normal mucosal tissue was examined as a reference sample. The specimens were obtained from the pathology department of the hospital. The initial histological evaluation was reconfirmed by pathologists (HT and MT) and the final diagnosis was classified as low-grade (LG) adenoma, high-grade (HG) adenoma, or carcinoma.

The study protocol of the clinical research was approved by the Asahikawa Medical University Research Ethics Committee and written informed consent was obtained from each patient (Approval No. 18169). This study was conducted in accordance with the principles of the Declaration of Helsinki. A written informed consent was obtained from each patient.

### Target sequencing with NGS

Mutation profiles were determined by target amplicon sequencing using NGS according to a previously reported method (Tanabe et al., 2020). Briefly, formalin-fixed paraffin embedded (FFPE) specimens were prepared as 10-μm thin slides, and genomic DNA was isolated from using a GeneRead DNA FFPE Kit (Qiagen, Manchester, UK). Ten serial sections were made, in which one of sections were stained with Hematoxylin and eosin (H & E). Objective lesions were marked on the stained slide and then corresponding areas in the serial unstained slides were micro-dissected for DNA purification. A colorectal cancer-associated gene panel was designed using the Ampliseq Designer Website; this panel consisted of 9 genes (*APC, KRAS*, *ARID1A*, *FBXW7*, *CTNNB1*, *BRAF*, *NRAS, MUTYH,* and *PIK3CA*) and 325 amplicons (Supplementary Table S1). Genomic DNA (20–60 ng) was amplified using this gene panel and a sequencing library was prepared. Sequencing and data analyses were performed using the GeneStudio S5 system (Thermo Fisher Scientific, Tokyo, Japan). Sequence reads were demultiplexed, quality-filtered, and aligned to the human reference genome (hg19/GRCh37) using the Torrent Suite software program and the Variant Caller plugin.

### Data analyses

To identify somatic mutations, the cutoff value of the NGS analysis was set to a variant allele frequency of 5%. *APC* mutations were plotted using Mutation Mapper (www.cbioportal.org) and the frequency of mutated *APC* genes in the MCR was calculated as the MCR rate. Copy number alteration (CNA) detection was performed using sequence data generated from the amplicon-based libraries, as previously reported (Aoki et al., 2020). The read counts of each amplicon were scaled using the total number of mapped reads for each primer pool in multiplex PCR of each sample. To evaluate the statistical significance of CNA for each region, a one-sample t-test was applied to the group of each neighboring amplicon within a gene from the normal copy number (Supplementary Material and Methods).

### Statistical analysis

Differences between groups were assessed using Pearson’s χ^2^ test or Fisher’s exact test for categorical variables and the Median test for continuous variables. We considered *P* < 0.05 to indicate statistical significance.

## Results

### Patient characteristics

**FIGURE 2:**
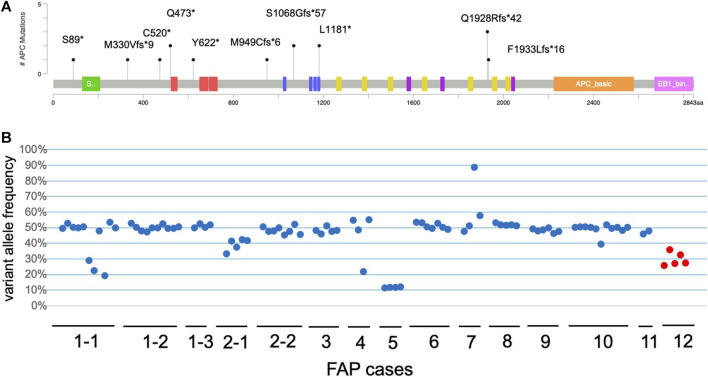
Distribution and variant allele frequency (VAF) of germline variants of *APC* in FAP patients. **(A)** Each lollipop represents the position of variants showing broad distribution in a gene of APC. Protein domains: green, oliomerization; red armadillo repeats; blue, 15 amino acid repeats; yellow, 20 amino acid repeats; purple, SAMP motif. **(B)** VAF of the germline variants of *APC* in the neoplastic lesions. Each blue dot represents VAF of each sample from FAP patients. Blue dots indicate VAFs from the patients with postzygotic mutation.

**FIGURE 3:**
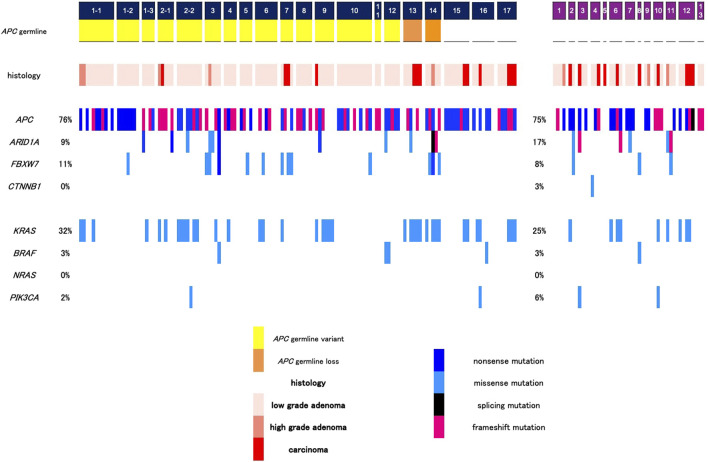
The somatic mutations in each neoplastic sample. Left panel, 20 familial adenomatous polyposis (FAP) patients in 17 pedigrees include 15 patients with *APC* germline variant, 2 with whole *APC* deletion, and 3 with no variants detected. Germ-line variants are confirmed by the analysis of non-neoplastic specimens. Right panel, 13 non-familial patients. Neoplastic polyps are used for the assessment of somatic mutations. The frequencies of pathogenic mutations of the genes are shown in the right side of the panels.

Twenty patients with polyposis were subjected to NGS analysis, and germline variants were identified using the reference sample. Twelve pedigrees, including 15 patients, showed germline *APC* pathogenic variants (Figure 2A). The germline variants in *APC* were listed in Table 1. FAP1 and 5 with germline *APC* variants (p.Q1928Rfs*42 and p.F1933Lfs*16, respectively) in the 3′ terminal and FAP11 with those (p.S89*) in the 5′ terminal were AFAP. The other nine pedigrees had FAP with sparse polyposis, and no profound type FAP was observed. Two cases were considered to have copy number loss, and further aCGH analysis confirmed the whole *APC* deletion in chromosome 5q (Supplementary Figure S1). Other three cases of polyposis did not show germline variants. Neither homozygous nor heterozygous *MUTYH* germline variants were found in any case. This NGS study included 20 patients with FAP, with 146 lesions, for which genetic and protein alteration were shown in Table 1. Ten patients underwent colectomy, whereas the others underwent endoscopic polypectomy. Genetic alterations in histologically confirmed LG adenomas (n = 102), HG adenomas (n = 5), and carcinoma (n = 13) are summarized in Figure 3. Twenty-two samples were dissected from normal mucosal lesions. Two sessile serrated lesions and two duplicate samples obtained from a single tumor were excluded from the analysis.

**TABLE 1:** Patient characteritics with germline APC mutations and treatments of the polyps.

No	Sex	APC germline	APC protein	Age	treatment	Polyps analysed	Total polyps	Low grade	High grade	Carcinoma
1-1	F	c.5782delC	p.Q1928Rfs*42	36	Total colectomy	11	11	9	2	​
1-2	M	c.5782delC	p.Q1928Rfs*42	19	Polypectomy	1	7	7	​	​
				20	Polypectomy	1				
				24	Polypectomy	5				
1-3	F	c.5782delC	p.Q1928Rfs*42	14	Polypectomy	2	4	4	​	​
				17	Polypectomy	1				
				19	Polypectomy	1				
2-1	F	c.1560_1563delCTCT	p.C520*	43	Hartmann	5	5	3	1	1
2-2	M	c.1560_1563delCTCT	p.C520*	23	Polypectomy	8	8	8	​	​
3	F	c.3202_3205delTCAA	p.S1068Gfs*57	20	Total colectomy	5	5	4	1	​
4	F	c.3202_3205delTCAA	p.S1068Gfs*57	28	Total colectomy	4	4	4	​	​
5	F	c.5799_5800delTCinsAAA	p.F1933Lfs*16	31	Total colectomy	4	4	4	​	​
6	F	c.1866C>A	p.Y622*	39	Polypectomy	1	7	7	​	​
				40	Polypectomy	1				
				42	Polypectomy	2				
				45	Polypectomy	3				
7	F	c.3542T>G	p.L1181*	57	Polypectomy	1	4	2	​	2
				58	Polypectomy	1				
				59	Polypectomy	2				
8	F	c.3542T>G	p.L1181*	31	Polypectomy	3	5	5	​	​
				31	Polypectomy	2				
9	F	c.2844delT	p.M949Cfs*6	34	Polypectomy	3	6	5	​	1
				34	Polypectomy	3				
10	F	c.988_989delAT	p.M330Vfs*9	38	Polypectomy	11	11	11	​	​
11	F	c.266C>A	p.S89*	53	Polypectomy	2	2	2	​	​
12	M	c.1417C>T	p.Q473*	31	Total colectomy	5	5	5	​	​
13	M	Loss		43	Total colectomy	6	6	3	​	3
14	F	Loss		32	Polypectomy	2	5	4	1	​
				32	Polypectomy	3				
15	M	Not detected		72	Right hemi colectomy	8	8	6	​	2
16	F	Not detected		39	Total colectomy	7	7	6	​	1
17	F	Not detected		63	Total colectomy	6	6	3	​	3

A total of 51 lesions from 13 patients with non-polyposis colonic adenomatous polyps were subjected to genetic analysis (Figure 1). The following lesions were included in the study: LG adenomas (n = 23), HG adenomas (n = 3), and carcinomas (n = 10). Ten samples dissected from normal lesions adjacent to the tumors were used as references, and sessile serrated lesions and traditional serrated adenomas were excluded from subsequent analyses.

The clinicopathological characteristics of polyps in FAP and sporadic cases were summarized in Table 2. FAP patients were significantly younger than sporadic cases, with median ages of 36 and 74 years, respectively (*P* < 0.001). Gender distribution also differed (*P* = 0.001); FAP cases were predominantly female, whereas sporadic cases were more frequently male. Although the location of the lesions (proximal, distal, or rectal) did not significantly differ between the two groups (*P* = 0.102), histological grade showed a marked difference (*P* = 0.015). Low-grade adenomas were more frequent in FAP (85.0%) than in sporadic cases (63.9%).

**TABLE 2:** Cliniclpathological chracteristics of the polyps according to the number of APC hits (somatic and germline variant).

Characteristics	Number of APC hits in FAP cases (%)					P-value	Number of APC hits in sporadic cases (%)					P-value	FAP vs. sporadic, P-value	
	Total	0	1	2	3		Total	0	1	2	3		Total number	0-3 hits
Age, years	​	​	​	​	​	0.171	​	​	​	​	​	0.459	<0.001	​
Median	36	39	38	34	36	​	74	74	72	75	76	​	​	​
Range	19-72	32-43	20-72	19-72	20-53	​	17-78	62-78	17-77	47-77	76	​	​	​
Gender	​	​	​	​	​	0.407	​	​	​	​	​	0.010	0.001	​
Male	34	2 (1.7)	8 (6.7)	23 (19.2)	1 (0.8)	​	24	3 (8.3)	13 (36.1)	7 (19.4)	1 (2.8)	​	​	0.038
Female	86	4 (3.3)	28 (23.3)	46 (39.3)	8 (6.7)	​	12	8 (22.2)	3 (8.3)	1 (2.8)	0 (0.0)	​	​	<0.001
Location	​	​	​	​	​	0.803	​	​	​	​	​	0.809	0.102	​
Proximal colon	37	2 (1.7)	13 (10.8)	18 (15.0)	4 (3.3)	​	15	6 (16.7)	6 (16.7)	3 (8.3)	0 (0.0)	​	​	0.010
Distal colon	41	3 (2.5)	11 (9.2)	24 (20.0)	3 (2.5)	​	18	5 (13.9)	8 (22.2)	4 (11.1)	1 (2.8)	​	​	0.047
Rectum	26	1 (0.8)	11 (9.2)	12 (10.0)	2 (1.7)	​	3	0 (0.0)	2 (5.6)	1 (2.8)	0 (0.0)	​	​	0.903
Unknown	16	0	1	15	0	​	0	0	0	0	0	​	​	​
Differentiation	​	​	​	​	​	0.503	​	​	​	​	​	0.520	0.015	​
Low grade adenoma	102	5 (4.2)	29 (24.2)	60 (50.0)	8 (6.7)	​	23	6 (16.7)	12 (33.3)	4 (11.1)	1 (2.8)	​	​	<0.001
High grade adenoma	5	0 (0.0)	3 (2.5)	2 (1.7)	0 (0.0)	​	3	2 (5.6)	1 (2.8)	0 (0.0)	0 (0.0)	​	​	0.168
Carcinoma	13	1 (0.8)	4 (3.3)	7 (5,8)	1 (0.8)	​	10	3 (8.3)	3 (8.3)	4 (11.1)	0 (0.0)	​	​	0.490
Total	120	6 (5.0)	36 (30.0)	69 (57.5)	9 (7.5)	​	36	11 (30.6)	16 (44.4)	8 (22.2)	1 (2.8)	​	​	<0.001

### Somatic mutations determined with NGS

**FIGURE 4:**
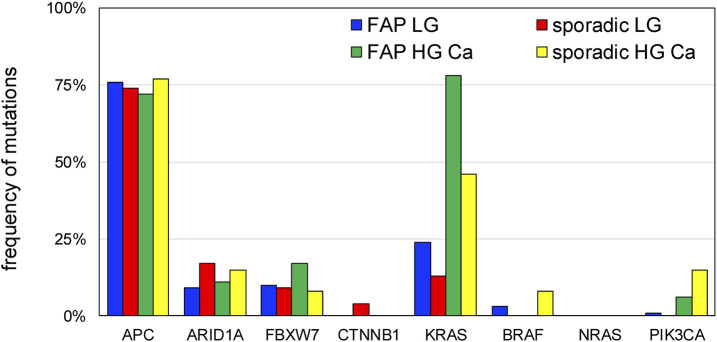
Mutation frequencies in the adenoma-carcinoma sequence. The frequencies of mutations are compared between low-grade adenomas and high-grade adenomas/carcinomas. LG, low-grade; HG, high-grade; Ca, carcinoma.

Landscapes of genomic alterations in FAP and sporadic tumors are shown in Figure 3. In all tumor samples from FAP patients with an *APC* germline variant, somatic mutations in the *APC* were frequently observed (76%). The frequency of *APC* mutation in LG adenomas was 72%, while that in HG adenomas and carcinomas was 72% (Figure 4). The majority of these mutations are nonsense or frameshift mutations that cause amino acid truncation. Insertion and/or deletion of nucleotides were found in 28 (46.7%), and nonsense mutations were found in 31 (51.7%) of 60 FAP polyps. Other alterations include single-nucleotide substitutions. The major mutation signatures of single-nucleotide changes in *APC* were C > T and C > A substitutions. *KRAS* mutations were found in 32% of the FAP samples (24% of LG adenomas and 78% of HG adenomas and carcinomas), while *BRAF* mutations were found in only 3% of all samples (serrated tumors were excluded from the analysis). *KRAS* mutations are observed in patients: FAP2-2, 9, 13, and 14. *ARID1A* mutations were observed in FAP3, and *FBXW7* mutations were found in FAP3, 7, and 14. These mutations clustered in an intra-patient manner.

*APC* mutations were found in 75% of sporadic colonic adenomatous polyps and carcinomas that we examined, while *KRAS* mutations were found in 25% of cases. The frequency of *KRAS* mutations in HG adenomas and carcinomas (6/13, 46%) was significantly higher than that in LG adenomas (3/23, 13%).

### Distribution of somatic APC mutations

**FIGURE 5:**
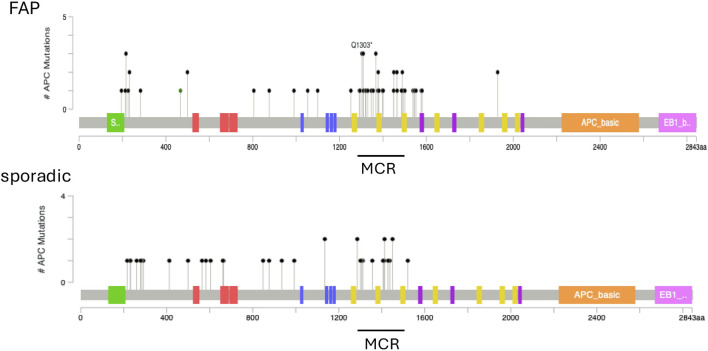
Somatic mutations in the *APC* gene. The position of *APC* mutations is represented by lollipop charts. Top, familial adenomatous polyposis (FAP) patients. A high proportion of mutations occurs within the mutation cluster region (MCR; codon 1286–1513). Bottom, non-familial patients with sporadic tumors. Most of the mutations are found throughout the first 1600 codons of the *APC* genes.

The somatic mutations found in the adenomatous polyps of FAP and non-polyposis patients are plotted in Figure 5. Mutant loci in *APC* appeared to be more widely distributed in the sporadic colorectal neoplasms than in neoplasms of FAP patients. Somatic *APC* mutations confined to the MCR between codons 1,286 and 1,513 were found in 63.3% (38/60) of polyps from the FAP patients and in 44.4% (16/36) of the polyps from without patients.

### Distribution of germline variants and somatic mutations in the FAP patients

**FIGURE 6:**
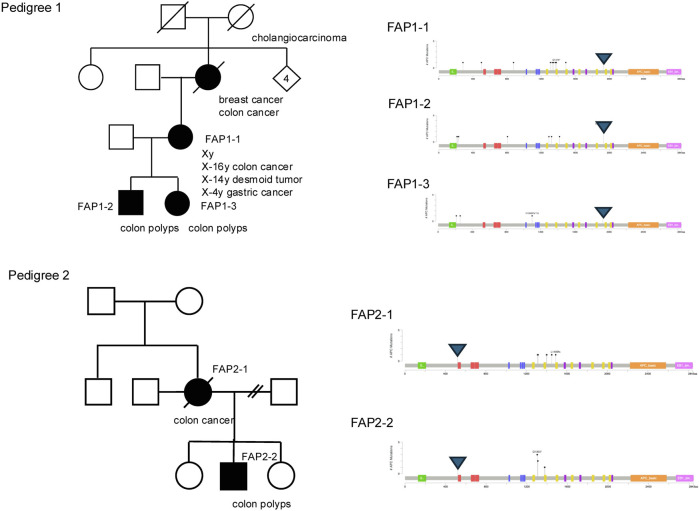
Family trees and the position of germline and somatic *APC* gene alteration. The lollipop charts indicate the positions of somatic *APC* alterations of each patient The triangles indicate the genetic variants of Family 1 (top) and 2 (bottom). Similar distribution patterns of alterations are observed in a pedigree.

Polyps from FAP cases showed a distinct number of *APC* hits compared with sporadic cases (Table 2). In FAP, many polyps (57.5%) harbored two *APC* hits, whereas in sporadic cases, one or two hits were more common (44.4% and 22.2%, respectively). Overall, the total number of *APC* mutational hits differed significantly between FAP and sporadic polyps (*P* < 0.001). Ten germline pathogenic variants from 12 FAP pedigrees and whole *APC* deletion in two patients were identified, for whom 99 colorectal polyps were analyzed for association between germline and somatic mutations. We searched for an association between germline and somatic mutations based on the number of 20AARs remaining in both the mutant alleles. The somatic mutations were located heterogeneously, retaining 0-3 20AARs (Table 3). Five patients in two pedigrees have similar pattern of somatic mutations indicating association between a germline variant and somatic mutations (Figure 6). Somatic *APC* mutations were observed in 74.7% (74/99) of the polyps, and double somatic mutations were observed in 9.1% (9/99) of polyps. Nine tumors from FAP patients therefore possessed triple alterations, including a germline variants and double somatic mutations (Supplementary Figure S2). An FAP patient with a whole *APC* deletion and double somatic mutations was identified. In non-polyposis patients with 36 sporadic colorectal polyps, single somatic mutations and double mutations were observed in 44.4% (16/36) and 22.2% (8/36) of patients, respectively.

**TABLE 3:** Association between germline and somatic variants in FAP polyps.

Germline	20AARs	Somatic				
		0	1	2	3	Total
5'-	0	12	17	15	8	52
3'-	5	11	8	2	1	22
Whole APC deletion	0	0	0	9	0	9

### Copy number alteration in the APC gene

Copy number loss was observed in two patients with polyposis who had no germline *APC* variants in any sample tissue. *APC* loss was observed in the normal reference as well as in each polyp of FAP13 and FAP14 (Supplementary Figure S3). CNA was also examined in terms of the change in variant allele frequency (VAF) of the germline *APC* variants (Figure 2B). Most of the germline variants presented around 50% VAF in the tumor tissues as well as reference samples. Increased VAF was observed in a polyp of FAP7 and decreased VAFs in polyps of FAP1-1, 2-1, 4, 5, and 12. Among them, tumors in FAP12 showing a decrease in VAF of approximately 30% (25.6%–35.7%) was confirmed a VAF of 16.7% in the blood material, and he was then diagnosed with postzygotic mutation. In somatic *APC* mutations, VAFs varied in each sample and distributed less than 50% (Supplementary Table S2).

## Discussion

We investigated early genetic events associated with colorectal carcinogenesis using NGS. Our genetic analysis indicated a high frequency of *APC* somatic mutations in adenomas of both FAP patients and sporadic cases. *APC* mutations were observed in 75% of the colorectal adenomas, and the frequency of the mutation was sustained through the adenoma-carcinoma sequences (Figure 4). Thus, *APC* mutations are most likely associated with the initiation of colorectal adenoma. Peterson et al. has developed a mathematical model of colorectal cancer initiation and demonstrated that the order of the driver genes was *APC* loss as the first event followed by *KRAS* gain in large majority of cases (Paterson et al., 2020). The frequency of mutation of colorectal cancer was compared to the genetic alterations deposited in the database (Supplementary Figure S4). *APC* mutations are the most frequently observed mutations, and *KRAS* mutations are considered major cancer-related genetic mutations. Early carcinogenesis in our FAP patients was similar to that in sporadic cases when these genetic alterations were compared in our study. The late phase of colorectal carcinogenesis may be regulated by MAPK signaling, such as *KRAS* and *BRAF*, but not by WNT signaling. Another carcinogenic process (e.g., for mucinous carcinoma) is regulated by a *p53* mutation without an *APC* mutation. We further showed that *KRAS* mutations were commonly identified in HG adenoma and early carcinoma of FAP patients. A previous study on FAP tumors suggested that *KRAS* mutations were not necessary in *APC*-associated FAP adenomas, as codon 12 mutations are rarely (10%) observed (Obrador-Hevia et al., 2010; Bettington et al., 2013). A later study detected *KRAS* mutations in 23% of FAP tumor samples and somatic *APC* mutations in 69% of the patients (Li et al., 2020). These discrepancies may have depended on the dysplasia grade in these studies, as the previous study included many small adenomas. Among our FAP patients, the frequency of *KRAS* mutations in LG adenomas was 24%, which corresponds to the rate reported in the previous study. The comparison between FAP-associated and sporadic neoplasia clearly demonstrated that the MAPK pathway is associated with carcinogenesis in some FAP patients. Colorectal tumors with *KRAS* mutations were frequent in some FAP patient, and the positive rates differed among the patients. Subsequently, we propose intra-patient homogeneity and inter-patient heterogeneity in genetic mutations of colorectal tumors in FAP.

There is a clinical question of whether *APC* mutations contribute to the progression of advanced CRC. The frequency of *APC* mutations in carcinomas, which has been investigated in a large number of studies in TCGA, is 51% in hypermutated tumors and 81% in non-hypermutated tumors. Truncated APC protein is thus expected to be critical for the initiation of benign colorectal adenomas, but not for the promotion of malignant lesions. *APC* mutations were recently reported to be associated with a good prognosis (Mondeca et al., 2020), whereas *KRAS* and *BRAF* mutations were reported to be associated with later carcinogenesis and poor prognosis in patients with CRC (Walther et al., 2009; Pai et al., 2012). In our FAP cohort, the frequency of *KRAS* mutation in patients with HG adenomas or carcinomas was higher than that in patients with sporadic tumors. The disease severity of colorectal tumors in FAP and sporadic tumors may vary depending on the metabolic process or proliferative activity (Li et al., 2020). Carcinogenicity differs among patients because *KRAS* mutations are frequently observed in polyps of some patients and not in others. Remarkably, polyps in the FAP patients with whole *APC* deletion (FAP13 and FAP14) frequently possessed *KRAS* mutations (5/6 and 4/5, in Figure 3). The reason for the intra-patient homogeneity in oncogene mutations should be investigated further. *APC* loss establishes the adenomatous phenotype, while *KRAS* activation enhances proliferation, reduces apoptosis, and facilitates invasion. The adenoma-carcinoma sequence may be accelerated in some patients through genetic and environmental changes including bacterial colonization.

The germline *APC* variants were not located within a particular region in the 15 FAP patients, with variant located at the 5′ terminal (S89* in FAP11) and two at the 3′-terminal (Q1928Rfs* in FAP1, F1933Lfs* in FAP5). Whereas *APC* somatic mutations in the MCR were frequently observed in polyps of our FAP patients. In contrast, *APC* mutations in sporadic adenomas were more widely distributed in the 5′- half of the gene. Mutations in this region have been reported to maintain 1 or 2 of the 20AARs, controlling the ability to bind β-catenin, which translocates into the nucleus and activates transcriptional factors in the Wnt signaling pathway (Aitchison et al., 2020). The ‘just-right’ signaling model explains these observations. In our analysis of attenuated FAP patients, in whom germline pathogenic variants were in the 5′ and 3′ terminals, the somatic mutations were located heterogeneously, retaining 0-3 20AARs. The loose-fit hypothesis was clearly adopted. The number of preserved 20AARs is quite different in sporadic colorectal cancer (Christie et al., 2013). In contrast, FAP patients with whole *APC* deletion (FAP13 and 14) had somatic mutations in MCR retaining 2 20AARs. The correlation between germline variant and somatic mutations is associated with genotype-phenotype correlation.

One of the important discoveries in this study was the three-hit *APC* alteration, which was detected in nine polyps from seven FAP patients. They retain the first hit in the germline, and the second and/or third hits occurs in the somatic region (Sieber et al., 2006). Judging from the locations of the second and third hits, we could not identify any rules. The three-hit hypothesis that mutations and polyploidy cause multiple changes in a single cancer cell was proposed in 2009 (Segditsas et al., 2009). Subsequently, many types of genetic alterations were detected by NGS analysis because it is more sensitive than Sanger sequencing. Ultrasensitive genotyping of *APC* mutations using nanofluidic PCRs clearly demonstrates intra-crypt heterogeneity (Gausachs et al., 2017; Garcia et al., 1999). Therefore, the second and third hits were considered to occur at separate crypt levels. The heterogeneity of tumor cells in a single polyp may produce multiple crypts with a few hits in FAP carcinogenesis.

The present study has some technical limitations. First, the genetic alterations analyzed in this study were limited. The whole-exome sequence of the mutational signature was investigated in a recent study (Aitchison et al., 2020) Because FFPE specimens were used for genetic analysis, short-read sequencing was performed. Long-read sequencing may provide additional information regarding the zygosity of the three-hits. Second, epigenetic alterations have not yet been investigated. The most common possible epigenetic change (i.e., gene methylation) frequently reduces proteins (Sieber et al., 2006). Transcriptomic profiling of carcinogenesis requires fresh tissues from patients.

## Conclusion

Through NGS analysis of colorectal neoplasms, we identified the genetic profile of the colonic adenoma-carcinoma sequence and the high prevalence of *APC* somatic mutations in colorectal tumors from both FAP patients and sporadic cases. A combined analysis of germline and somatic alterations revealed ‘three-hit’ alterations in the *APC* gene among FAP patients, suggesting that the heterogeneity of colorectal carcinogenesis contribute to these genetic changes. The observed correlation between germline pathogenic variants and somatic mutations resembles a genotype-phenotype relationship. Further structural analyses are needed to elucidate the mechanisms of tumor development in colorectal polyps of FAP patients.

## Data Availability

All relevant data of the next-generation sequencing are within the Supplementary Table S2. Novel variant data are publicly available in the International Society of Gastrointestinal Hereditary Tumors (InSiGHT) variant database (https://www.insight-group.org/variants/databases/): APC c.1560_1563delCTCT (FAP2), #0000873264; APC c.5799_5800delTCinsAAA (FAP5), #0001021155; APC c.3542C>A (FAP8), #0001021156; APC c.2844delT (FAP9), #0001021157.

## Supplementary material

SUPPLEMENTARY FILE 1 Supplementary Materials and Methods.

SUPPLEMENTARY TABLE S1 Targeted regions of the genes explored by the AmpliSeq custom panel.

SUPPLEMENTARY TABLE S2 Table S2.

Supplementary Presentation 1 Supplementary Figures S1-S4.
