# Supplementary material for: Early genetic events in the colorectal carcinogenic pathway of familial adenomatous polyposis and sporadic polyp: germline and somatic alterations in carcinogenesis
Source: Front Genet. 2025 Dec 19;16:1668133. doi: 10.3389/fgene.2025.1668133 (PMC12757105; doi:10.3389/fgene.2025.1668133)
Supplement: Supplementary file 2 [file Supplementaryfile1.docx]

**Supplementary Materials and Methods**

Figure S3

An assessment of CNA detection in sequencing data generated from the amplicon-based libraries was conducted (Aoki 2020). First, the read counts of each amplicon were scaled with the total number of mapped reads for each primer pool of multiplex PCR of each sample, using a formula known as reads per million mapped reads (RPM). After logarithmic transformation of RPM+1 with base 2, the arithmetic means of 42 sequence data from histologically normal tissues was subtracted from the corresponding amplicon value for each pathological tissue sample. GC bias was then corrected except that the correction was carried out in the logarithmic axis, and the smoothness parameter of span = 0.7 was provided using the <loess> function of R (version 3.3.2; The R Foundation). Subsequently, a global normalization was performed by subtracting the median within each sample from all amplicon values. Finally, the normalized logarithmic values were reverse-transformed into the copy number ratio axis. For evaluating the statistical significance of CNA for each region, a one-sample t-test was applied to the group of each five neighboring amplicons within a gene from the normal copy number. CNAs are called when passing the significance threshold of p-value < 0.000029, where Bonferoni’s correction was applied to the threshold value.

Figure S4

We screened colorectal cancer (CRC) patients from the cBioPortal for Cancer Genomics (cBioPortal database, <https://www.cbioportal.org/>). The criteria for selecting the studies in cBioPortal database were: (a) studies containing CRC patients; (b) studies with detailed NGS results. Studies of “bowel” tissue were explored, and 10 studies were included, shown in Study of origin. Selected genomic profiles were Mutations, Structural Variant, and Copy Number. Nine genes of interest (*APC, KRAS*, *ARID1A*, *FBXW7*, *CTNNB1*, *BRAF*, *NRAS, MUTYH,* and *PIK3CA*) were entered to submit query. The data accession date was September 4, 2020, when the study protocol was designed.
