# Supplementary material for: Early genetic events in the colorectal carcinogenic pathway of familial adenomatous polyposis and sporadic polyp: germline and somatic alterations in carcinogenesis
Source: Front Genet. 2025 Dec 19;16:1668133. doi: 10.3389/fgene.2025.1668133 (PMC12757105; doi:10.3389/fgene.2025.1668133)
Supplement: Supplementary file 3 [file Presentation1.pptx]

## Slide 1
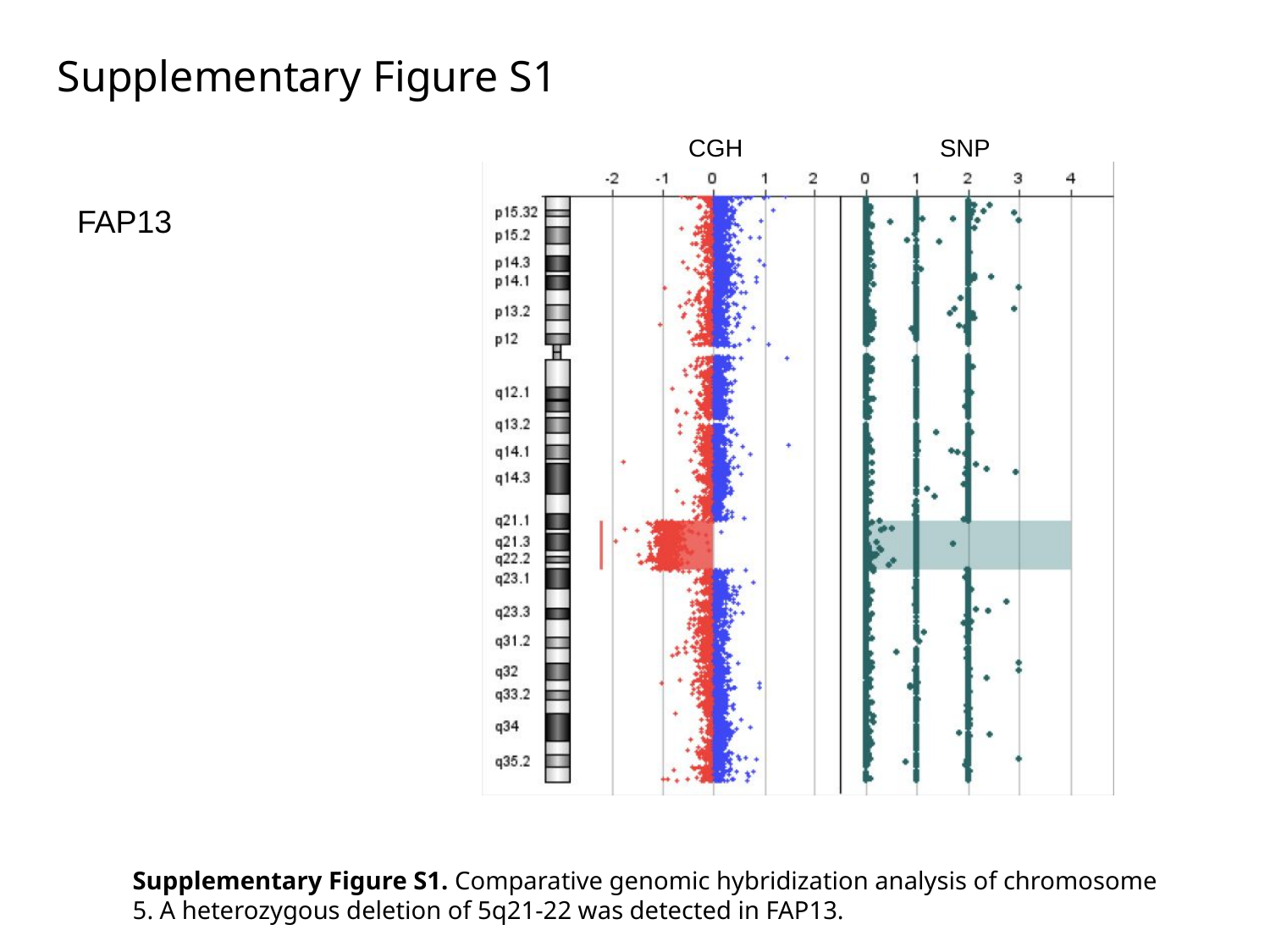

Supplementary Figure S1
CGH
SNP
 FAP13
Supplementary Figure S1. Comparative genomic hybridization analysis of chromosome 5. A heterozygous deletion of 5q21-22 was detected in FAP13.

## Slide 2
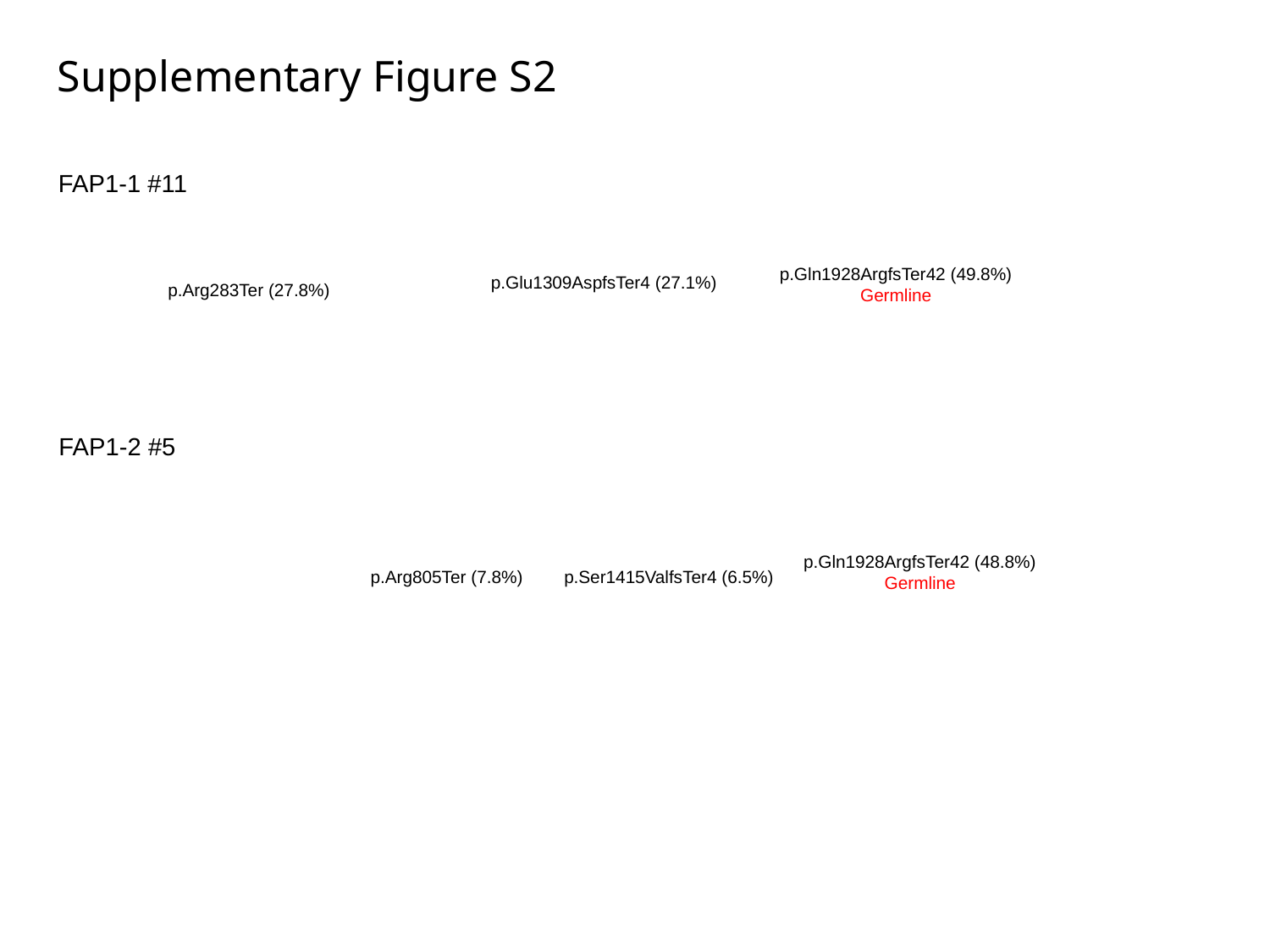

Supplementary Figure S2
FAP1-1 #11
p.Gln1928ArgfsTer42 (49.8%)
Germline
p.Glu1309AspfsTer4 (27.1%)
p.Arg283Ter (27.8%)
FAP1-2 #5
p.Gln1928ArgfsTer42 (48.8%)
Germline
p.Arg805Ter (7.8%)
p.Ser1415ValfsTer4 (6.5%)

## Slide 3
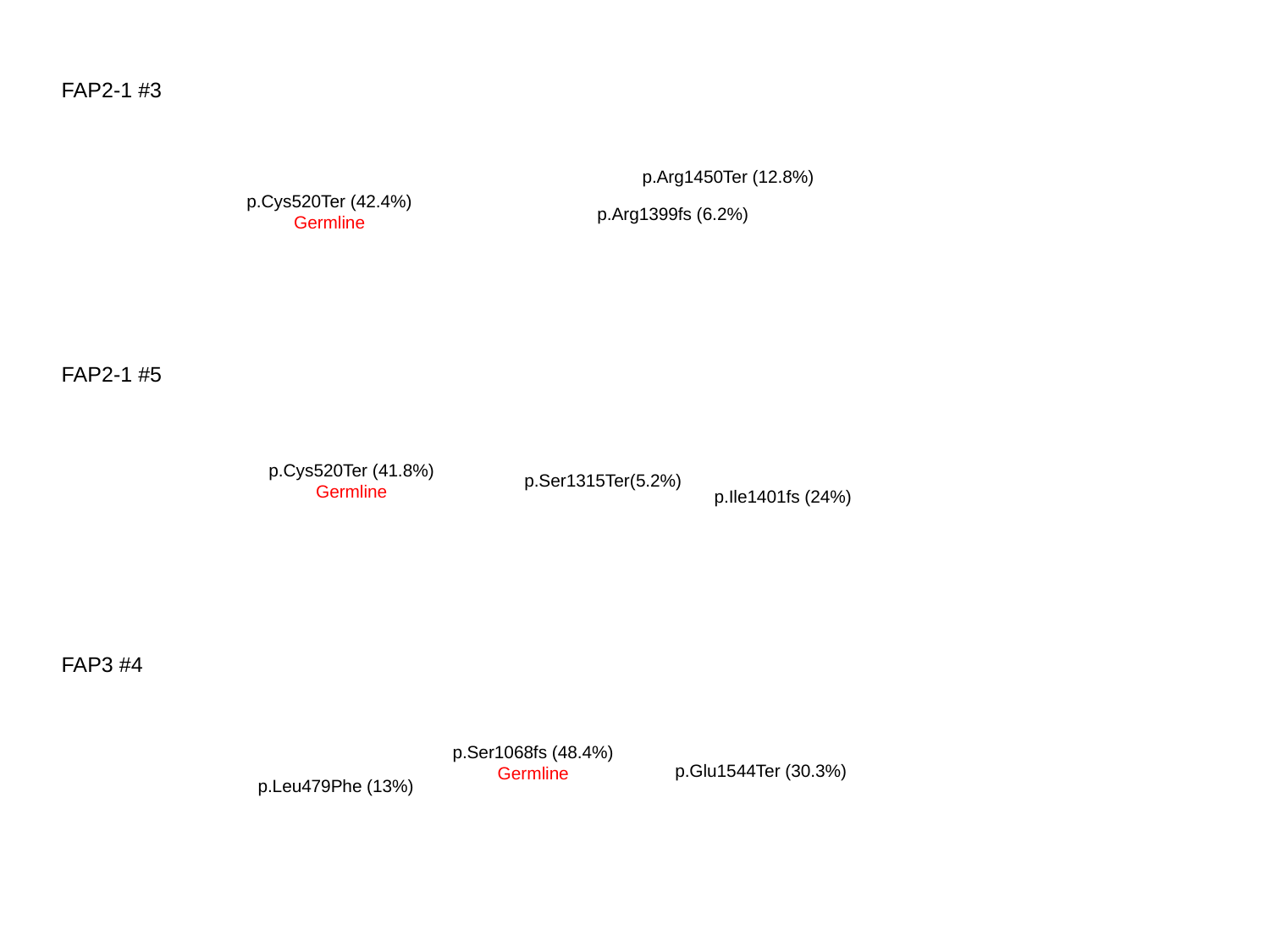

FAP2-1 #3
p.Arg1450Ter (12.8%)
p.Cys520Ter (42.4%)
Germline
p.Arg1399fs (6.2%)
FAP2-1 #5
p.Cys520Ter (41.8%)
Germline
p.Ser1315Ter(5.2%)
p.Ile1401fs (24%)
FAP3 #4
p.Ser1068fs (48.4%)
Germline
p.Glu1544Ter (30.3%)
p.Leu479Phe (13%)

## Slide 4
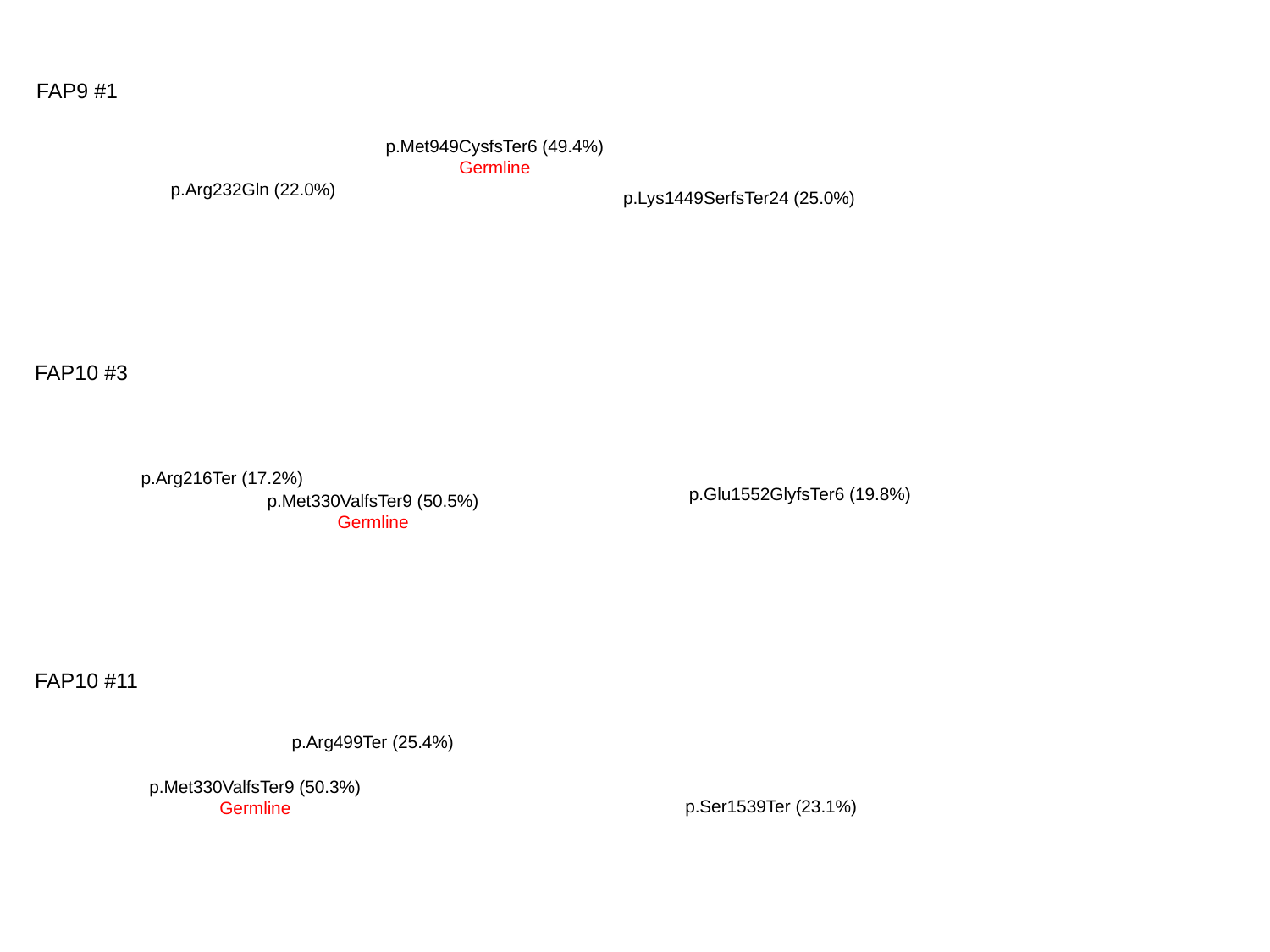

FAP9 #1
p.Met949CysfsTer6 (49.4%)
Germline
p.Arg232Gln (22.0%)
p.Lys1449SerfsTer24 (25.0%)
FAP10 #3
p.Arg216Ter (17.2%)
p.Glu1552GlyfsTer6 (19.8%)
p.Met330ValfsTer9 (50.5%)
Germline
FAP10 #11
p.Arg499Ter (25.4%)
p.Met330ValfsTer9 (50.3%)
Germline
p.Ser1539Ter (23.1%)

## Slide 5
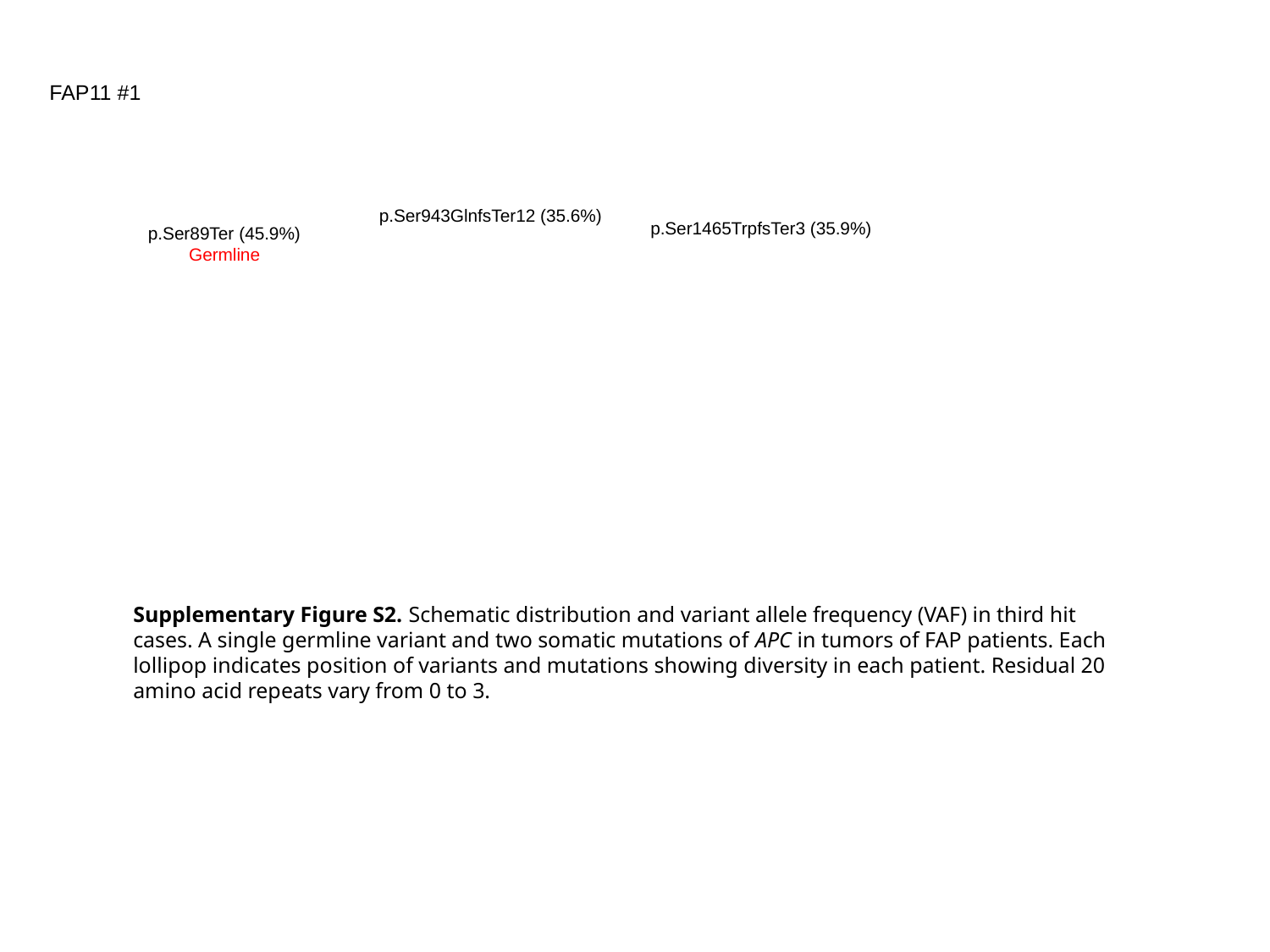

FAP11 #1
p.Ser943GlnfsTer12 (35.6%)
p.Ser1465TrpfsTer3 (35.9%)
p.Ser89Ter (45.9%)
Germline
Supplementary Figure S2. Schematic distribution and variant allele frequency (VAF) in third hit cases. A single germline variant and two somatic mutations of APC in tumors of FAP patients. Each lollipop indicates position of variants and mutations showing diversity in each patient. Residual 20 amino acid repeats vary from 0 to 3.

## Slide 6
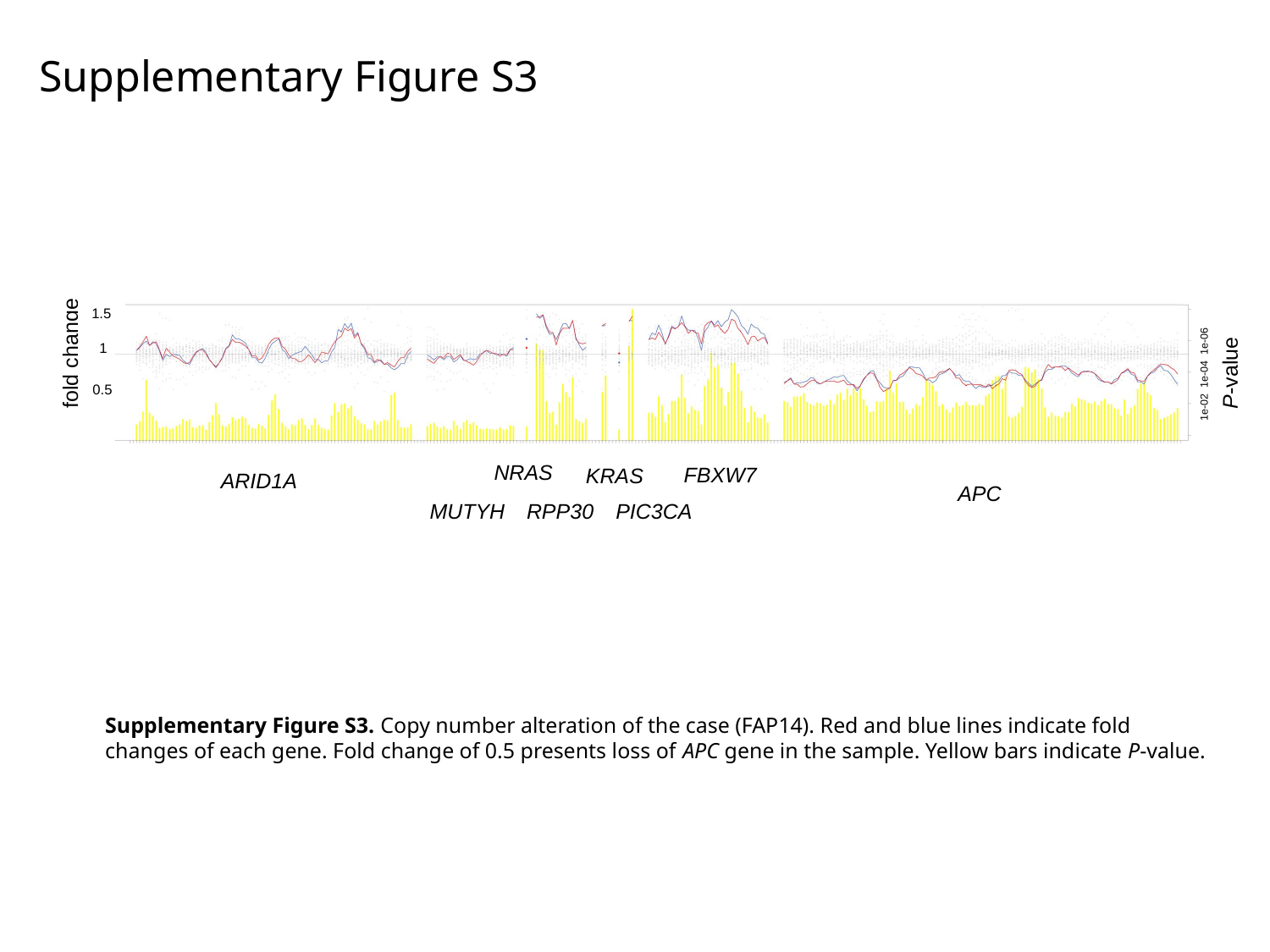

Supplementary Figure S3
1.5
1
fold change
P-value
1e-02 1e-04 1e-06
0.5
NRAS
FBXW7
KRAS
ARID1A
APC
MUTYH
RPP30
PIC3CA
Supplementary Figure S3. Copy number alteration of the case (FAP14). Red and blue lines indicate fold changes of each gene. Fold change of 0.5 presents loss of APC gene in the sample. Yellow bars indicate P-value.

## Slide 7
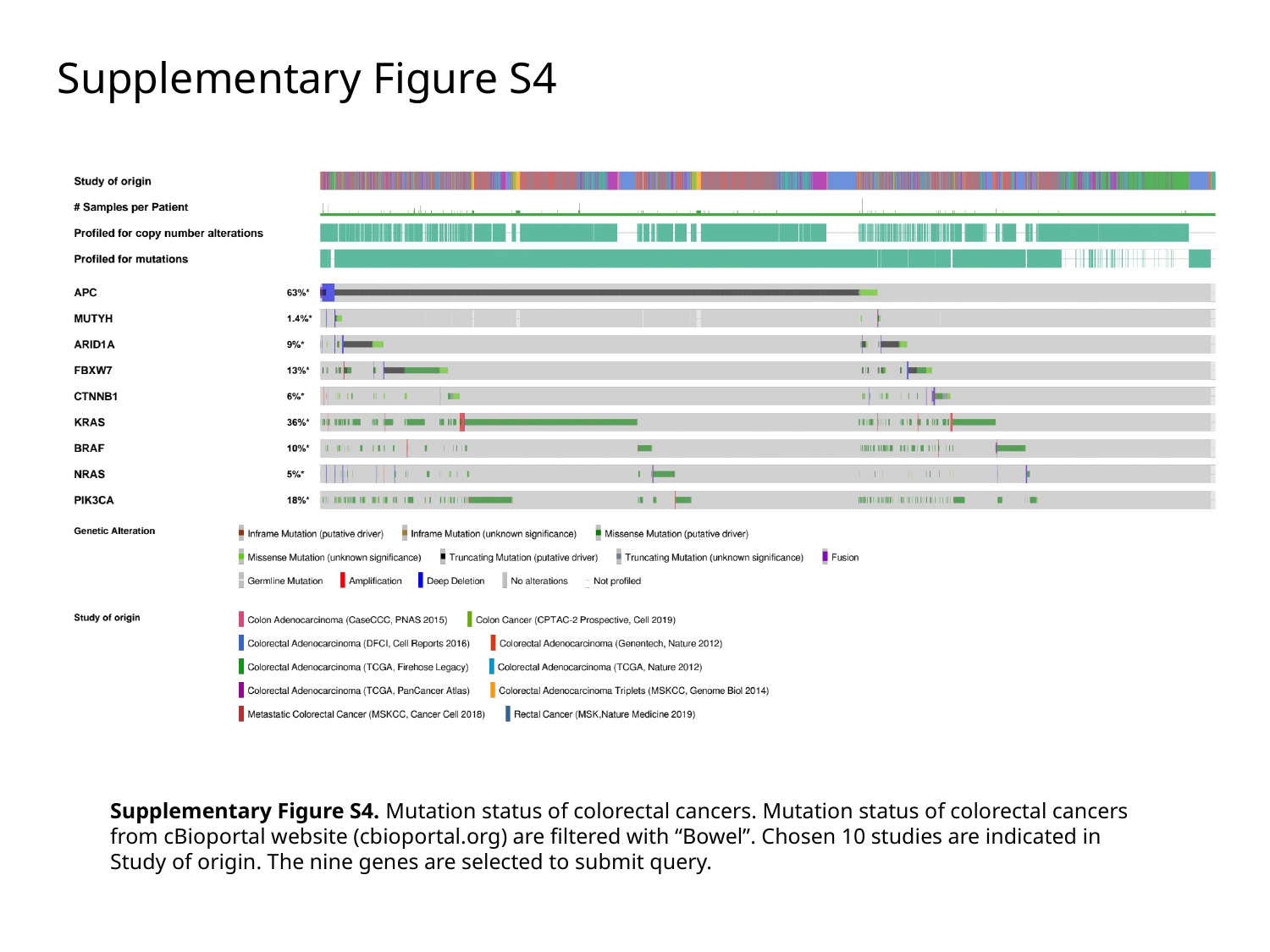

Supplementary Figure S4
Supplementary Figure S4. Mutation status of colorectal cancers. Mutation status of colorectal cancers from cBioportal website (cbioportal.org) are filtered with “Bowel”. Chosen 10 studies are indicated in Study of origin. The nine genes are selected to submit query.
